# Active contours driven by local and global fitted image models for image segmentation robust to intensity inhomogeneity

**DOI:** 10.1371/journal.pone.0174813

**Published:** 2017-04-04

**Authors:** Farhan Akram, Miguel Angel Garcia, Domenec Puig

**Affiliations:** 1 Department of Computer Engineering and Mathematics, Rovira i Virgili University, Tarragona, Spain; 2 Department of Electronic and Communications Technology, Autonomous University of Madrid, Madrid, Spain; Center for Neuroscience and Regenerative Medicine, UNITED STATES

## Abstract

This paper presents a region-based active contour method for the segmentation of intensity inhomogeneous images using an energy functional based on local and global fitted images. A square image fitted model is defined by using both local and global fitted differences. Moreover, local and global signed pressure force functions are introduced in the solution of the energy functional to stabilize the gradient descent flow. In the final gradient descent solution, the local fitted term helps extract regions with intensity inhomogeneity, whereas the global fitted term targets homogeneous regions. A Gaussian kernel is applied to regularize the contour at each step, which not only smoothes it but also avoids the computationally expensive re-initialization. Intensity inhomogeneous images contain undesired smooth intensity variations (bias field) that alter the results of intensity-based segmentation methods. The bias field is approximated with a Gaussian distribution and the bias of intensity inhomogeneous regions is corrected by dividing the original image by the approximated bias field. In this paper, a two-phase model is first derived and then extended to a four-phase model to segment brain magnetic resonance (MR) images into the desired regions of interest. Experimental results with both synthetic and real brain MR images are used for a quantitative and qualitative comparison with state-of-the-art active contour methods to show the advantages of the proposed segmentation technique in practical terms.

## Introduction

Image segmentation is an important stage in image processing and computer vision [1]. Intensity inhomogeneity is one of the well-known problems in image segmentation, which arises from the imperfections of the image acquisition process or due to external interferences. It manifests as a smooth intensity variation across the image that complicates the segmentation of the objects contained in it. For instance, in medical image analysis, segmentation and registration stages are highly sensitive to spurious variations of image intensity. Therefore, the complexity of intensity inhomogeneity can lead to false results and assumptions that can be critical for decision making by both doctors and radiologists. This is why numerous methods for intensity inhomogeneity correction have been proposed in the past [2].

Thus, intensity inhomogeneity correction is often a pre-processing stage necessary for achieving better image segmentation. In turn, correct segmentation makes intensity inhomogeneity correction rather trivial. Actually, both intensity inhomogeneity correction and segmentation can be viewed as two intertwined processes. In segmentation-based intensity inhomogeneity correction methods, both processes are merged such that they benefit from each other.

The techniques that aim to avoid intensity inhomogeneity in the image acquisition process are known as *prospective* *methods*. They are only capable of correcting intensity inhomogeneity caused by the imaging device, not being able to segment the objects affected by intensity inhomogeneity. On the other hand, techniques that can correct an image and also segment the objects affected by intensity inhomogeneity are called *retrospective* *methods*. Retrospective methods are further classified according to the image segmentation method they apply into: filtering methods [3, 4], surface fitting methods [5, 6], histogram-based methods [7, 8], and active contours [9–15]. Segmentation-based methods [10–12] are the most versatile, since they unify segmentation and bias correction in a single framework. In these methods, segmentation and bias correction are applied in conjunction to benefit from each other.

Active contours are retrospective methods suitable for both image segmentation and bias correction [10–12, 16–18]. The first active contour method was proposed in [19] in order to segment an image by evolving a curve towards the boundary of an object contained in the image. An energy functional is first defined by using image statistics, curvature and gradient information. The curve is evolved by minimizing that energy functional. Active contour methods can be categorized into edge-based [19–21] and region-based [22–36] methods.

Edge-based active contour methods typically use image gradient information to define an inflating balloon force that is used in the curve evolution process [20]. However, in case of intense noise or weak edges, edge-based active contours can hardly converge to the right contours. Therefore, edge-based methods are not suitable for that kind of images.

Alternatively, region-based active contour methods use statistical and curvature information from the image in the formulation of the energy functional. They can be further characterized into global [22–26] and local [27–34] methods. Mumford and Shah [22] devised a global region-based active contour method by assuming image homogeneity. Thus, traditional active contour methods based on [22], such as the *active contours without edges* (ACWE) method [23], cannot segment images with intensity inhomogeneity. These methods usually compute intensity averages over the whole image. Therefore, they cannot deal with small changes between distinct regions nor segment objects with weak or blurred boundaries. On the other hand, local-based methods [27, 28] are able to distinguish small changes between the background and the foreground. Therefore, they are suitable for intensity inhomogeneous images.

A region-based active contour model able to process image information in local regions was proposed by Li et al. in [27, 28]. The major contribution of that work was the introduction of a local binary fitting (LBF) energy with a kernel function that enables the extraction of accurate local image information. Therefore, that model can be used to segment images with intensity inhomogeneity, which overcomes the limitation of piecewise constant models [23].

**Fig 1:**
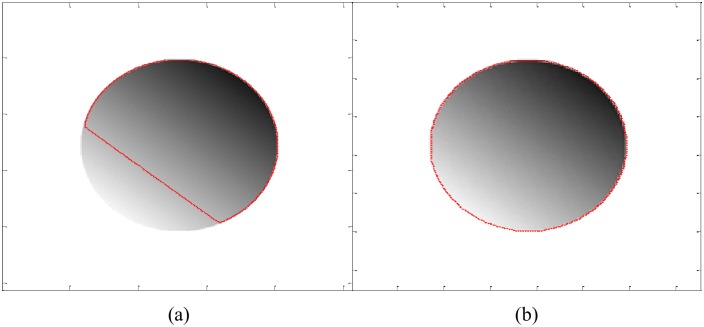
Intensity inhomogeneous image segmentatation using (a) ACWE and (b) LBF methods.

Fig 1(a) shows that a traditional region based active contour method (such as ACWE) is unable to segment an image with an intensity inhomogeneous object. However, a local active contour method (such as LBF) is able to properly segment such object as shown in Fig 1(b). Taking Fig 1 as a reference, it can be concluded that active contour methods which use local statistical information of an image are able to produce fairly acceptable segmentation results in the context of intensity inhomogeneity.

A region-based active contour model with a variational level-set formulation for image segmentation was presented in [29]. It uses local intensity means for computing the level-set curve. Local image intensities are described by local Gaussian distributions (LGD) to guide the motion of the contour toward the object boundaries. Therefore, it can be used to segment images in the presence of intensity inhomogeneity and noise. In particular, the model is able to distinguish regions with different intensity variances.

A local active contour model for segmenting images with intensity inhomogeneity was proposed by Zhang et al. in [30]. Local image information is used to define a local image fitting (LIF) energy functional, which can be interpreted as a constraint on the differences between the fitting image [27, 28] and the original image. Furthermore, a new method is used to regularize the level-set function by using Gaussian kernel filtering after each iteration. In addition, re-initialization of the level-set curve is not required.

In [25], a region-based active contour method is formulated by using a global *signed* *pressure* *force* (SPF) function in the energy function. The global SPF function is defined by using global intensity means from the ACWE model [23]. A Gaussian kernel is used to regularize the level-set and prevent re-initialization.

Alternatively, a region-based active contour method is formulated in [31] in the context of intensity inhomogeneity by utilizing local intensity means. It uses an SPF function based on a local fitted image in its energy formulation in order to segment images with intensity inhomogeneity. A Gaussian kernel is used to smooth the level-set function after every step. Therefore, this method does not require re-initialization.

A variational level-set approach for bias correction and segmentation (VLSBCS) for images corrupted with intensity inhomogeneity was proposed by Li et al. in [9, 10]. The computed bias field is intrinsically ensured to be smooth by the data term in the variational formulation, without any additional effect to maintain the smoothness of the bias field.

A local statistical active contour model (LSACM) for image segmentation in the presence of intensity inhomogeneity was proposed by Zhang et al. in [11, 12]. The inhomogeneous objects are modelled as Gaussian distributions of different means and variances, and a moving window is used to map the original image into a new domain in which the intensity distributions of inhomogeneous objects are still Gaussian but better separated. The means of the Gaussian distributions in the transformed domain can be adaptively estimated by multiplying the bias field with the original signal within the window. A statistical energy functional is then defined for each local region, which combines the bias field, the level-set function, and the constant approximating the true signal of the corresponding object.

The present paper proposes a new region-based active contour method that incorporates both local and global fitted images in the energy functional. The local term helps segment objects with intensity inhomogeneity, whereas the global term accelerates the evolution of the contour over smooth homogeneous regions. The new energy functional is formulated with the assumption that the local fitted image from [30] can be divided into two different local and global components. In the gradient descent solution of the proposed energy functional, both local and global signed pressure force (SPF) functions are introduced. The local SPF function is formulated by using local image differences and deals with inhomogeneous regions. In turn, the global SPF function is formulated by using the global image difference in order to segment homogeneous regions while reducing the convergence time of the level-set curve. The replacement of the local and global image differences by their respective SPF functions makes the gradient descent solution more stable. Finally, a Gaussian kernel is used to regularize the level-set curve, which also avoids the computationally expensive re-initialization. In this paper, the proposed two-phase model is also extended to a four-phase model in order to successfully segment a brain MR image into three regions: white matter (WM), grey matter (GM) and cerebrospinal fluid (CSF) regions, which is not possible with a two-phase model. Experiments with both synthetic and real images demonstrate that the proposed method yields better segmentation results and offers bias-corrected images with more detail than state-of-the-art methods.

This paper is organized as follows. The theoretical foundations are discussed in section 2. The proposed method is described in section 3. Experimental results and comparisons are shown in section 4 using both synthetic and real brain magnetic resonance images as a practical application scope. Quantitative analysis is presented in section 5 using a public database of brain anatomical models [37]. Finally, conclusions and further research lines are given in section 6.

## Theoretical foundations

### Mumford-Shah energy model

In the image segmentation problem, Mumford and Shah proposed an active contour method to find an optimum piecewise smooth approximation function *u* of an image. Let *I*: Ω → **R**^2^, where *u* varies within each sub-region Ω_*i*_ of the image domain smoothly, and rapidly or discontinuously across the boundaries of Ω_*i*_ in order to approximate a closed curve *C* ⊂ Ω along the object boundary. They proposed the following energy functional:

$$E_{{\;}_{MS}}=\lambda \int_{\Omega }|I\left(x\right)-u\left(x\right){|}^{2}\;dx+v\int_{\Omega \backslash C}|\nabla u\left(x\right){|}^{2}\;dx+\mu L\left(C\right),\;x\in \Omega$$ (1)

where *L*(*C*) is the length of the curve *C*, and *μ* and *v* ≥ 0 are fixed parameters. The unknown contour *C* and the non-convexity of the above energy functional make it difficult to minimize it. Some alternative methods were later proposed to simplify or modify the above functional, as described below.

### Chan-Vese model

Chan and Vese [23] proposed an active contour method based on the Mumford and Shah model [22]. Let *I*: Ω → **R**^2^ be an input image, *ϕ*: Ω → **R**^2^ a level set, and *C* a closed curve corresponding to the zero level set: *C* = {*x* ∈ Ω|*ϕ*(*x*) = 0}. The following energy functional is defined:

$$\begin{array}{ll} E_{CV} & =\lambda_{1}\underset{\Omega }{\int }\left|I\left(x\right)-m_{1}{|}^{2}H_{\epsilon }\left(\phi \right)dx+\lambda_{2}\underset{\Omega }{\int }\right|I\left(x\right)-m_{2}{|}^{2}\left(1-H_{\epsilon }\left(\phi \right)\right)dx\\ & +\mu \underset{\Omega }{\int }|\nabla H_{\epsilon }\left(\phi \right){|}^{2}dx+v\underset{\Omega }{\int }H_{\epsilon }\left(\phi \right)dx \end{array}$$ (2)

where *μ* ≥ 0, *v* ≥ 0, (*λ*_1_, *λ*_2_)>0 are fixed parameters and *H*_*ϵ*_(*ϕ*) is the regularized version of the Heaviside function:

$$H_{\epsilon }\left(\phi \right)=\frac{1}{2}+\frac{1}{\pi }arctan\left(\frac{\phi }{\epsilon }\right)$$ (3)

Parameter *ϵ* controls the smoothness of the Heaviside function. For *ϵ* → 0, the Heaviside function is the ideal unit step function. Parameter *μ* scales the Euclidian length of the curve *C* in (Eq 2), which is used to regularize the contour. In turn, parameter *v* scales the area term in (Eq 2), which is used to compute the area of the region inside *C*. Constants *m*_1_ and *m*_2_ approximate the image intensities inside and outside contour *C*, respectively. By minimizing the above energy functional with respect to *ϕ* through steepest gradient descent [38], the following gradient descent flow is obtained:

$$\begin{array}{ll} \frac{\partial \phi }{\partial t} & =(-\lambda_{1}{\left(I\left(x\right)-m_{1}\right)}^{2}+\lambda_{2}{\left(I\left(x\right)-m_{2}\right)}^{2}\\ & +\mu \text{ div}\left(\frac{\nabla \phi }{\left|\nabla \phi \right|}\right)-v)\delta_{\epsilon }\left(\phi \right), \end{array}$$ (4)

where *δ*_*ϵ*_(*ϕ*) is the regularized Dirac function:

$$\delta_{\epsilon }\left(\phi \right)=\frac{\epsilon }{\pi (\phi^{2}+\epsilon^{2})}$$ (5)

In addition to specifying the smoothness of the Heaviside function (3), parameter *ϵ* also controls the width of the Dirac function. For *ϵ*→ 0, the Dirac function is the ideal unit impulse. By minimizing (Eq 2) with respect to *m*_1_ and *m*_2_ while keeping *ϕ* constant, *m*_1_ and *m*_2_ are defined as:

$$\begin{array}{ll} m_{1} & =\frac{\int_{\Omega }I\left(x\right)H_{\epsilon }\left(\phi \right)dx}{\int_{\Omega }H_{\epsilon }\left(\phi \right)dx},\\ m_{2} & =\frac{\int_{\Omega }I\left(x\right)\left(1-H_{\epsilon }\left(\phi \right)\right)dx}{\int_{\Omega }\left(1-H_{\epsilon }\left(\phi \right)\right)dx} \end{array}$$ (6)

The data fitting term − *λ*_1_(*I* − *m*_1_)^2^ + *λ*_2_(*I* − *m*_2_)^2^ in (Eq 4) plays a key role in the curve evolution. Parameters *λ*_1_ and *λ*_2_ weight the first and the second term, respectively. In most cases, *λ*_1_ = *λ*_2_ and *v* = 0 when the image is smooth and the signal-to-noise ratio is low. Parameter *μ* is a scale factor. If it is low enough, small objects are likely to be extracted. Alternatively, if it is high, big objects can be detected [23]. Obviously, *m*_1_ and *m*_2_ in (Eq 6) are related to the global properties of the image contents inside and outside curve *C*, respectively. However, such a global image segmentation is not accurate if the image intensity inside and/or outside the curve is inhomogeneous.

### Local Binary Fitted (LBF) model

Li et al. [27, 28] proposed the LBF model by embedding local image information in the energy functional. LBF is able to segment images with intensity inhomogeneity. The basic idea is to introduce a Gaussian kernel function to define the LBF energy functional as follows:

$$\begin{array}{ll} E_{LBF} & =\lambda_{1}\int_{\Omega }K_{\sigma }\left(x-y\right)|I\left(y\right)-f_{1}\left(x\right){|}^{2}H_{\epsilon }\left(\phi \right)dydx\\ & +\lambda_{2}\int_{\Omega }K_{\sigma }\left(x-y\right)|I\left(y\right)-f_{2}\left(x\right){|}^{2}\left(1-H_{\epsilon }\left(\phi \right)\right)dydx, \end{array}$$ (7)

where (*λ*_1_, *λ*_2_) > 0 are fixed parameters, *I*: Ω → **R**^2^ is an input image, *K*_*σ*_ is a Gaussian kernel with standard deviation *σ*, and *f*_1_ and *f*_2_ are two smooth functions that approximate the local image intensities inside and outside curve *C*, respectively.

In LBF, *C* ⊂ Ω can be represented by the zero level-set of a Lipschitz function *ϕ*: Ω ⊂ **R**. Minimizing the energy functional *E*_*LBF*_ with respect to *ϕ*, the gradient descent flow is defined as follows:

$$\frac{\partial \phi }{\partial t}=-\left(\lambda_{1}e_{1}-\lambda_{2}e_{2}\right)\delta_{\epsilon }\left(\phi \right),$$ (8)

where *e*_1_ and *e*_2_ are defined as follows:

$$\begin{array}{ll} e_{1}\left(x\right) & =\underset{\Omega }{\int }K_{\sigma }\left(x-y\right)|I\left(y\right)-f_{1}\left(x\right){|}^{2}dy,\\ e_{2}\left(x\right) & =\underset{\Omega }{\int }K_{\sigma }\left(x-y\right)|I\left(y\right)-f_{2}\left(x\right){|}^{2}dy \end{array}$$ (9)

In (Eq 8), parameters *λ*_1_ and *λ*_2_ weight the two integrals over the regions inside and outside curve *C*, respectively, which are defined in (Eq 9). In most cases, *λ*_1_ = *λ*_2_. Functions *f*_1_ and *f*_2_ are the local intensity means inside and outside curve *C*, which are computed in a local neighbourhood:

$$\begin{array}{ll} f_{1}\left(x\right) & =\frac{K_{\sigma }*\left[I\left(x\right)H_{\epsilon }\left(\phi \right)\right]}{K_{\sigma }*H_{\epsilon }\left(\phi \right)},\\ f_{2}\left(x\right) & =\frac{K_{\sigma }*\left[I\left(x\right)\left(1-H_{\epsilon }\left(\phi \right)\right)\right]}{K_{\sigma }*\left(1-H_{\epsilon }\left(\phi \right)\right)} \end{array}$$ (10)

Functions *f*_1_ and *f*_2_ in (Eq 10) represent weighted averages of image intensities in a Gaussian window inside and outside the curve, respectively. In that way, the LBF model can handle images with intensity inhomogeneity.

The standard deviation *σ* of the Gaussian kernel plays an import role in practical applications. It behaves as a scale parameter that controls the region-scalability from the small neighbourhood to the whole image domain [28]. It must be properly chosen according to the images. A too small *σ* may cause an undesirable result, whereas a too large *σ* will yield a high computational cost.

In order to guarantee a stable evolution of the level-set function, the distance regularized term defined in [21] is incorporated into (Eq 8). Moreover, the Euclidean length term is used to regularize the zero contour of *ϕ*. Finally, the total variational formulation is as follows:

$$\begin{array}{ll} \frac{\partial \phi }{\partial t} & =\gamma \left(\nabla^{2}\phi -\text{div}\left(\frac{\nabla \phi }{\left|\nabla \phi \right|}\right)\right)\\ & +\left(\mu \text{ div}\left(\frac{\nabla \phi }{\left|\nabla \phi \right|}\right)-\left(\lambda_{1}e_{1}-\lambda_{2}e_{2}\right)\right)\delta_{\epsilon }\left(\phi \right), \end{array}$$ (11)

where *γ* is a scaling parameter of the distance regularized energy penalization term that controls the energy leakage and *μ* is a scaling parameter of the Euclidian length of the curve.

### Local Image Fitted (LIF) model

In [30], a local image fitting energy functional is proposed in which the difference between the fitted image and the original image is minimized as follows:

$$E_{LIF}=\frac{1}{2}\underset{\Omega }{\int }|I\left(x\right)-I_{LFI}\left(x\right){|}^{2}dx,$$ (12)

where *I*_*LFI*_ is the local fitted image defined as:

$$I_{LFI}\left(x\right)=f_{1}\left(x\right)M_{1}+f_{2}\left(x\right)M_{2},$$ (13)

where *M*_1_ = *H*_*ϵ*_(*ϕ*), *M*_2_ = (1 − *H*_*ϵ*_(*ϕ*)), and *f*_1_ and *f*_2_ are local intensity means in the given image (Eq 10). *H*_*ϵ*_(*ϕ*) is the regularized version of the Heaviside function (3). Using the calculus of variations and steepest gradient descent [38], *E*_*LIF*_ in (Eq 12) is minimized with respect to *ϕ* to yield the corresponding gradient descent flow:

$$\frac{\partial \phi }{\partial t}=\left(I\left(x\right)-I_{LFI}\left(x\right)\right)\left(f_{1}\left(x\right)-f_{2}\left(x\right)\right)\delta_{\epsilon }\left(\phi \right),$$ (14)

where *δ*_*ϵ*_(*ϕ*) is the regularized Dirac function (11).

### Variational Level-Set approach for Bias Correction and Segmentation (VLSBCS)

In [9, 10], a variational level-set method for the segmentation and bias correction of images corrupted with intensity inhomogeneity is formulated. In this method, the computed bias field is ensured to be smooth exclusively through the data term defined in the energy functional formulation. This method is based on an image model commonly used to describe images with intensity inhomogeneity:

I(x)=b(x)J(x)+n(x), (15)

where *I*(*x*) is the input image with intensity inhomogeneity, *J*(*x*) is the image to be restored without intensity inhomogeneity, *b*(*x*) is the bias field, which represents the modulation of the restored image with the intensity inhomogeneity, and *n*(*x*) is noise. The model assumes that the restored image *J*(*x*) is constant within each object in the image, i.e., $J\left(x\right)\approx \sum_{i=1}^{N}c_{i}M_{i}$ for *x*∈ Ω_*i*_, with $x\in {\left\{\Omega_{i}\right\}}_{i=1}^{N}$ being a sub-region of Ω.

In traditional active contour methods, the image domain Ω is assumed to be divided into *N* disjoint regions, Ω_*i*_, *i* = 1, 2, …., *N*, based on the input image *I*(*x*). However, due to the intensity inhomogeneity caused by the bias field *b*(*x*), the measured intensities are not separable by using traditional intensity-based segmentation methods.

In [9, 10], a K-means clustering method based on the minimization of the following objective function is proposed:

$$E≅\int \left(\sum_{i=1}^{N}\int_{\Omega_{i}}K_{\sigma }\left(x-y\right)|I\left(y\right)-b\left(x\right)c_{i}{|}^{2}dy\right)dx,$$ (16)

where *b*(*x*) is the approximated bias field and *c*_*i*_ is the computed intensity mean in the presence of intensity inhomogeneity for each of the phases of the two-phase active contour method (*i* = 1, 2). Term *b*(*x*)*c*_*i*_ can be considered to be the approximation of the means *m*_*i*_ of the clusters corresponding to each of the phases of the two-phase active contour method: *m*_*i*_ ≈ *b*(*x*)*c*_*i*_.

Directly minimizing the above energy functional with the partition ${\left\{\Omega_{i}\right\}}_{i=1}^{N}$ as a variable is not feasible. Therefore, multiple level-set functions are used to represent a partition ${\left\{\Omega_{i}\right\}}_{i=1}^{N}$. In the simplest case of *N* = 2, the image domain is partitioned into two regions {Ω_1_, Ω_2_}. These regions are separated by the zero-level contour of a function *ϕ*, that is, Ω_1_ ≅ {*ϕ* > 0} and Ω_2_ ≅ {*ϕ*< 0}. Using the Heaviside function *H*_*ϵ*_, the energy *E* in (Eq 16) becomes:

$$E=\int \left(\sum_{i=1}^{N}\int_{\Omega }K_{\sigma }\left(x-y\right)|I\left(y\right)-b\left(x\right)c_{i}{|}^{2}M_{i}\left(\phi \right)dy\right)dx,$$ (17)

where *M*_*i*_ is the characteristic function of a given image based on the regularized Heaviside function (3). When the image domain is partitioned in two regions {Ω_1_, Ω_2_}, *M*_*i*_ is defined as *M*_1_ = *H*_*ϵ*_(*ϕ*) and *M*_2_ = (1 − *H*_*ϵ*_(*ϕ*)). By taking the Gateaux derivative (the first order functional derivative) [38] of the energy *E*, the following expressions for *b*(*x*) and *c*_*i*_ are obtained:

$$b\left(x\right)=\frac{\sum_{i=1}^{2}K_{\sigma }*\left[I\left(x\right)c_{i}M_{i}\left(\phi \right)\right]}{\sum_{i=1}^{2}K_{\sigma }*\left[c_{i}^{2}M_{i}\left(\phi \right)\right]},$$ (18)

$$c_{i}=\frac{\int K_{\sigma }*\left[I\left(x\right)b\left(x\right)M_{i}\left(\phi \right)\right]dx}{\int K_{\sigma }*\left[b^{2}\left(x\right)M_{i}\left(\phi \right)\right]dx},$$ (19)

For *N* = 4, the image is partitioned into four regions (Ω_1_, Ω_2_, Ω_3_, Ω_4_) using two level sets *ϕ*_1_ and *ϕ*_2_. Then, *M*_*i*_ is defined as *M*_1_(Φ) = *H*_*ϵ*_(*ϕ*_1_)*H*_*ϵ*_(*ϕ*_2_), *M*_2_(Φ) = *H*_*ϵ*_(*ϕ*_1_) (1 − *H*_*ϵ*_(*ϕ*_2_)), *M*_3_(Φ) = (1 − *H*_*ϵ*_(*ϕ*_1_))*H*_*ϵ*_(*ϕ*_2_) and *M*_4_(Φ) = (1 − *H*_*ϵ*_(*ϕ*_1_)) (1 − *H*_*ϵ*_(*ϕ*_2_)).

### Local Statistical Active Contour Model (LSACM)

In order to segment and correct bias in an intensity inhomogeneous image a local statistical active contour model is devised in [11, 12], in which the inhomogeneous objects are modelled as Gaussian distributions of different means and variances. The means of the Gaussian distributions are adaptively estimated by multiplying the bias field by the original signal within a Gaussian window. Let *I*: Ω → **R**^2^ be the input image, Φ: Ω → **R**^2^ a level set function, which yields a closed curve *C* = {*x*∈ Ω|Φ(*x*) = 0}, *b*(*x*) is the approximated bias field, *c*_*i*_and *σ*_*i*_are intensity means and variances, respectively. Φ is a function of one level set *ϕ* for a two-phase segmentation and Φ is a function of two level sets (*ϕ*_1_, *ϕ*_2_) for four-phase segmentation. Based on the above assumptions, an energy functional is defined as:

$$E\left(\Phi \right)=\int_{\Omega }K_{\beta }\left(x,y\right)\left(log\left(\sigma_{i}\right)+{\left(I\left(y\right)-b\left(x\right)c_{i}\right)}^{2}/2\sigma_{i}^{2}\right)M_{i}\left(\Phi \right)dx,\;N=2\;or\;N=4$$ (20)

where *K*_*β*_(*x*, *y*) is a Gaussian kernel with a standard deviation *β*. For *N* = 2, the energy functional in (Eq 20) acts as a two-phase active contour method (with one level set). The two characteristic terms are: *M*_1_(Φ) = *H*_*ϵ*_(*ϕ*) and *M*_2_(Φ) = 1 − *H*_*ϵ*_(*ϕ*)). In turn, for *N* = 4, the energy functional in (Eq 20) acts as a four-phase active contour method (with two level sets *ϕ*_1_and *ϕ*_2_). The four characteristic terms are defined as: *M*_1_(Φ) = *H*_*ϵ*_(*ϕ*_1_)*H*_*ϵ*_(*ϕ*_2_), *M*_2_(Φ) = *H*_*ϵ*_(*ϕ*_1_) (1 − *H*_*ϵ*_(*ϕ*_2_)), *M*_3_(Φ) = (1 − *H*_*ϵ*_(*ϕ*_1_))*H*_*ϵ*_(*ϕ*_2_) and *M*_4_(Φ) = (1 − *H*_*ϵ*_(*ϕ*_1_))*H*_*ϵ*_(*ϕ*_2_) *H*_*ϵ*_(*ϕ*_2_)). In (Eq 20), bias correction *b*(*x*), intensity means *c*_*i*_ and variance *σ*_*i*_ are defined as:

$$b\left(x\right)=\frac{\sum_{i=1}^{N}K_{\beta }*\left(IM_{i}\left(\Phi \left(x\right)\right)\right)\cdot \frac{c_{i}}{\sigma_{i}^{2}}}{\sum_{i=1}^{N}K_{\beta }*M_{i}\left(\Phi \left(x\right)\right)\cdot \frac{c_{i}^{2}}{\sigma_{i}^{2}}}$$ (21)

$$c_{i}=\frac{\int \left(K_{\beta }*b\right)I\left(x\right)M_{i}(\phi (x))dx}{\int \left(K_{\beta }*b^{2}\right)M_{i}(\phi (x))dx,}$$ (22)

$$\sigma_{i}=\sqrt{\frac{\int \int \;K_{\beta }\left(x,y\right){\left(I\left(x\right)-b\left(x\right)c_{i}\right)}^{2}M_{i}(\phi \left(x\right))dydx}{\int K_{\beta }\left(x,y\right)M_{i}(\phi \left(x\right))dydx}}$$ (23)

## Proposed method

The generally accepted assumption on intensity inhomogeneity is that it manifests itself as a smooth spatially varying function that alters image intensities that otherwise would be constant for a same object regardless its position in an image. In its simplest form, the model assumes that intensity inhomogeneity is multiplicative or additive, that is, the intensity inhomogeneity field multiplies or adds to the image intensities. Most frequently, the multiplicative model has been used as it is consistent with the inhomogeneous sensitivity of the reception coil of magnetic resonance imaging devices. Therefore, a multiplicative model has been considered for the bias field estimation. Let *I*: Ω → **R**^2^ be the input image with intensity inhomogeneity, *J*(*x*) the restored image without intensity inhomogeneity, *b*(*x*) the bias field approximated with a Gaussian distribution, and *n*(*x*) additive noise (Eq 15).

*J*(*x*) is assumed to be constituted by *k* piecewise constant image components. *I*(*x*) can thus be represented as:

$$I\left(x\right)=b\left(x\right)\left\{c_{1}M_{1}+c_{2}M_{2}+......+c_{k}M_{k}\right\},$$ (24)

where *c*_*i*_ are intensity means computed for the piecewise regions ${\left\{\Omega_{i}\right\}}_{i=1}^{N}$ and *M*_*i*_ is the characteristic function of each region.

In order to segment intensity inhomogeneous images, the following energy functional is defined:

$$E_{g,LGFI}=E_{LGFI}(\phi )+\mu L_{g}(\phi )+vA_{g}\left(\phi \right),$$ (25)

where *E*_*LGFI*_ (*ϕ*) is a term based on a local and global fitted image, which will be explained later in this section. *μ* ≥ 0 and *v* ≥ 0 are fixed parameters. *L*_*g*_(*ϕ*) and *A*_*g*_(*ϕ*) are length and area terms, respectively [21]:

$$L_{g}\left(\phi \right)=\int_{\Omega }g\left(I\right)\delta_{\epsilon }\left(\phi \right)\left|\nabla \phi \right|dx,$$ (26)

$$A_{g}\left(\phi \right)=\int_{\Omega }g\left(I\right)H_{\epsilon }\left(-\phi \right)dx,$$ (27)

The energy functional *A*_*g*_(*ϕ*) is introduced to speed up the curve evolution. It is the area of the region $\Omega_{\phi }^{-}=\{\left(x,y\right)\left|\phi \left(x,y\right)<0\right\}$ [21]. *A*_*g*_(*ϕ*) can be viewed as the weighted area of $\Omega_{\phi }^{-}$. In (Eq 27), *g*(*I*) is a positive monotonously decreasing edge indicator function ranging in [0, 1]:

$$g\left(I\right)=\frac{1}{1+|\nabla K_{\sigma }{*I|}^{2}}$$ (28)

*E*_*LGFI*_ is defined according to the following reformulation of (Eq 12):

$$E_{LGFI}=\int_{\Omega }\left(I\left(x\right)-I_{bLFI}\left(x\right)\right)\left(I\left(x\right)-I_{GFI}\left(x\right)\right)dx,$$ (29)

### Two-phase active contours formulation

In (Eq 29), let *I*_*bLFI*_(*x*) be a two-phase bias local fitted image and *I*_*GFI*_(*x*) a global fitted image, using a level set *ϕ*, which are defined as:

$$I_{bLFI}\left(x\right)=b\left(x\right)\left(c_{1}M_{1}+c_{2}M_{2}\right),$$ (30)

$$I_{GFI}\left(x\right)=m_{1}M_{1}+m_{2}M_{2},$$ (31)

where *c*_1_and *c*_2_ are local intensity means and *m*_1_and *m*_2_ are global intensity means of the given image as defined in Eqs (19) and (6), respectively. *M*_1_ = *H*_*ϵ*_(*ϕ*) and *M*_2_ = (1 − *H*_*ϵ*_(*ϕ*)), where *H*_*ϵ*_(*ϕ*) is the regularized Heaviside function (3). Models based on global intensity means are not sufficient to solve intensity inhomogeneity segmentation problems. In turn, models based on local intensity means have a very high time complexity. Using both local and global fitted images, the proposed method is able to tackle the intensity inhomogeneity problem with a reduced time complexity.

As discussed earlier, an energy functional only based on a global fitted image cannot segment images with intensity inhomogeneity since global intensity means are computed under the assumption that the input image is homogeneous. Therefore, the bias field *b*(*x*) from (Eq 18) is only introduced in the local fitted image difference.

By using calculus of variations and steepest gradient descent [38], *E*_*LGFI*_ in (Eq 29) is minimized with respect to *ϕ*, leading to the corresponding gradient descent flow (refer to the appendix for a detailed derivation):

$$\begin{array}{ll} \frac{\partial \phi }{\partial t} & =(\left(I\left(x\right)-I_{bLFI}\left(x\right)\right)\left(m_{1}-m_{2}\right)\\ & +b\left(x\right)\left(I\left(x\right)-I_{GFI}\left(x\right)\right)\left(c_{1}-c_{2}\right))\delta_{\epsilon }\left(\phi \right), \end{array}$$ (32)

where *b*(*x*) is the bias field defined in (Eq 18), and {*m*_1_, *m*_2_} and {*c*_1_, *c*_2_} are global and local intensity means defined in Eqs (6) and (19), respectively. The above gradient descent flow is not stable around object boundaries. Moreover, it does not yield proper segmentation when the boundaries between inhomogeneous objects and the background are undistinguishable. (*I* − *I*_*bLFI*_) (*m*_1_ − *m*_2_) and (*I* − *I*_*GFI*_) (*f*_1_ − *f*_2_) generate large values that cross a maximum threshold resulting in an unstable contour. Therefore, (*I* − *I*_*bLFI*_) and (*I* − *I*_*GFI*_) are replaced by local and global signed pressure force (SPF) functions, which normalize values to [-1,1] to obtain a smooth version of the gradient descent flow:

$$\frac{\partial \phi }{\partial t}=\left(\lambda_{1}L_{SPF}\left(m_{1}-m_{2}\right)+\lambda_{2}bG_{SPF}\left(c_{1}-c_{2}\right)\right)\delta_{\epsilon }\left(\phi \right),$$ (33)

where the proposed local and global SPF functions are defined as:

$$\;\;L_{SPF}\left(I\right)=\left\{\begin{array}{cc} \frac{I\left(x\right)-I_{bLFI}\left(x\right)}{\;\text{max}\left(|I\left(x\right)-I_{bLFI}\left(x\right)|\right)}, & \;I(x)\neq 0\\ 0, & \;I(x)=0 \end{array}\right.$$ (34)

$$\;\;G_{SPF}\left(I\right)=\left\{\begin{array}{cc} \frac{I\left(x\right)-I_{GFI}\left(x\right)}{\;\text{max}\left(|I\left(x\right)-I_{GFI}\left(x\right)|\right)}, & \;I(x)\neq 0\\ 0, & \;I(x)=0 \end{array}\right.$$ (35)

By using the calculus of variations and steepest gradient descent, the solution of *E*_*g*,*LGFI*_ from (Eq 25) using Eqs (26) and (27) is:

$$\begin{array}{ll} \frac{\partial \phi }{\partial t} & =(\lambda_{1}L_{SPF}\left(m_{1}-m_{2}\right)+\lambda_{2}bG_{SPF}\left(c_{1}-c_{2}\right)\\ & +\mu \text{ div}\left(g\frac{\nabla \phi }{\left|\nabla \phi \right|}\right)+vg)\delta_{\epsilon }\left(\phi \right) \end{array}$$ (36)

The two scaling parameters *λ*_1_and *λ*_2_in Eqs (33) and (36) are used to tune the model to different types of images.

### Four-phase active contours formulation

In (Eq 29), let *I*_*bLFI*_(*x*) be a four-phase bias local fitted image and *I*_*GFI*_(*x*) a global fitted image, using two level sets Φ(*ϕ*_1_, *ϕ*_2_), which are defined as:

$$I_{bLFI}\left(x\right)=b\left(x\right)\left(c_{1}M_{1}+c_{2}M_{2}+c_{3}M_{3}+c_{4}M_{4}\right),$$ (37)

$$I_{GFI}\left(x\right)=m_{1}M_{1}+m_{2}M_{2}+m_{3}M_{3}+m_{4}M_{4}$$ (38)

where *M*_1_(Φ) = *H*_*ϵ*_(*ϕ*_1_)*H*_*ϵ*_(*ϕ*_2_), *M*_2_(Φ) = *H*_*ϵ*_(*ϕ*_1_) (1 − *H*_*ϵ*_(*ϕ*_2_)), *M*_3_(Φ) = (1 − *H*_*ϵ*_(*ϕ*_1_))*H*_*ϵ*_(*ϕ*_2_) and *M*_4_(Φ) = (1 − *H*_*ϵ*_(*ϕ*_1_)) (1 − *H*_*ϵ*_(*ϕ*_2_)), which are the characteristic terms that partition a given image into four segments. *b*(*x*) is the bias term defined for the four-phase energy functional. *c*_1_, *c*_2_, *c*_3_ and *c*_4_ are local intensity means from VLSBCS [9, 10] and *m*_1_, *m*_2_, *m*_3_and *m*_4_ are global intensity means from the multiphase level set framework (MLSF) [24], which is a multiphase extension of the two-phase energy functional defined by Chan-Vese [23].

By substituting the four-phase fitted equations in (Eq 29) using steepest gradient descent [38] the following solutions are obtained for *ϕ*_1_ and *ϕ*_2_ (for detailed formulation see appendix B):

$$\begin{array}{ll} \frac{\partial \phi_{1}}{\partial t} & =[b\left(I-I_{GFI}\right)\left(\left(c_{1}-c_{3}\right)H_{\epsilon }\left(\phi_{2}\right)+\left(c_{2}-c_{4}\right)\left(1-H_{\epsilon }\left(\phi_{2}\right)\right)\right)\\ & +\left(I-I_{bLFI}\right)\left(\left(m_{1}-m_{3}\right)H_{\epsilon }\left(\phi_{2}\right)+\left(m_{2}-m_{4}\right)\left(1-H_{\epsilon }\left(\phi_{2}\right)\right)\right]\delta_{\epsilon }\left(\phi_{1}\right) \end{array}$$ (39)

$$\begin{array}{ll} \frac{\partial \phi_{2}}{\partial t} & =[b\left(I-I_{GFI}\right)\left(\left(c_{1}-c_{2}\right)H_{\epsilon }\left(\phi_{1}\right)+\left(c_{3}-c_{4}\right)\left(1-H_{\epsilon }\left(\phi_{1}\right)\right)\right)\\ & +\left(I-I_{bLFI}\right)\left(\left(m_{1}-m_{2}\right)H_{\epsilon }\left(\phi_{1}\right)+\left(m_{3}-m_{4}\right)\left(1-H_{\epsilon }\left(\phi_{1}\right)\right)\right]\delta_{\epsilon }\left(\phi_{2}\right) \end{array}$$ (40)

Four-phase local and global SPF functions can be obtained by substituting *I*_*bLFI*_ and *I*_*GFI*_ from Eqs (37) and (38) in Eqs (34) and (35), respectively. By replacing the four-phase local and global fitted differences with their respective SPF functions, Eqs (39) and (40) are updated as follows:

$$\begin{array}{ll} \frac{\partial \phi_{1}}{\partial t} & =[\lambda_{1}bG_{SPF}\left(\left(c_{1}-c_{3}\right)H_{\epsilon }\left(\phi_{2}\right)+\left(c_{2}-c_{4}\right)\left(1-H_{\epsilon }\left(\phi_{2}\right)\right)\right)\\ & +\lambda_{2}L_{SPF}\left(\left(m_{1}-m_{3}\right)H_{\epsilon }\left(\phi_{2}\right)+\left(m_{2}-m_{4}\right)\left(1-H_{\epsilon }\left(\phi_{2}\right)\right)\right]\delta_{\epsilon }\left(\phi_{1}\right) \end{array}$$ (41)

$$\begin{array}{ll} \frac{\partial \phi_{2}}{\partial t} & =[\lambda_{1}bG_{SPF}\left(\left(c_{1}-c_{2}\right)H_{\epsilon }\left(\phi_{1}\right)+\left(c_{3}-c_{4}\right)\left(1-H_{\epsilon }\left(\phi_{1}\right)\right)\right)\\ & +\lambda_{2}L_{SPF}\left(\left(m_{1}-m_{2}\right)H_{\epsilon }\left(\phi_{1}\right)+\left(m_{3}-m_{4}\right)\left(1-H_{\epsilon }\left(\phi_{1}\right)\right)\right]\delta_{\epsilon }\left(\phi_{2}\right) \end{array}$$ (42)

**Fig 2:**
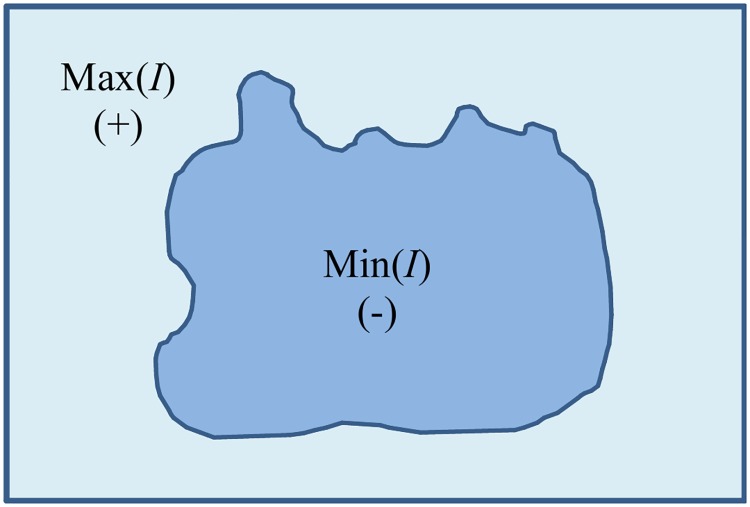
Signs of a global SPF function. It is positive outside the object and negative within it.

**Fig 3:**
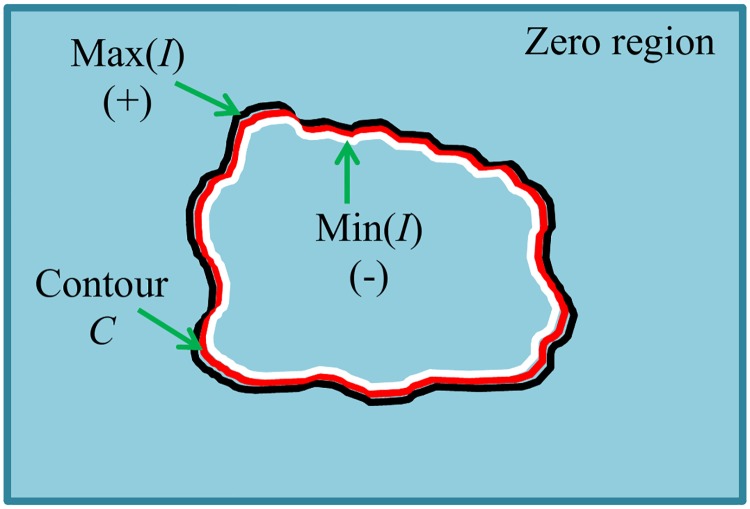
Signs of a local SPF function. It is positive along the outer boundary, negative along the inner boundary and zero elsewhere.

The SPF functions defined in Eqs (34) and (35) are used to normalize the local and global image differences in the range [-1, 1] inside and outside the region of interest. SPF functions have been formulated in numerous ways (e.g., [25, 26, 31, 36]), some of them incorporating global intensity means and others using local intensity means. The new SPF functions proposed in this work are based on both global and local intensity-based fitted images. Fig 2 shows the signs of an SPF function based on a global fitted image inside and outside the region of interest. In turn, Fig 3 shows the sign of an SPF function based on a local fitted image inside and outside the region of interest.

Since the local SPF function uses local intensity means, it is able to distinguish small changes at the boundaries of intensity inhomogeneous objects. Since the local intensity means are computed in a local neighbourhood, the positive and negative values are distributed close to the inner and outer boundaries of the image contours. In the rest of the image, the SPF function becomes zero. In Fig 3, white shows negative values of the local SPF function, black shows positive values of the local SPF function, and blue represents the region where the local SPF function becomes zero.

In contrast, the global SPF function is unable to segment intensity inhomogeneous regions, since global intensity means are computed all over the image. It has positive and negative values inside and outside the region of interest, and is zero at its boundary, as shown in Fig 2.

In level-set methods, it is essential to initialize the level-set function *ϕ* as a signed distance function (SDF) *ϕ*_0_. If the initial level-set function is significantly different from the SDF, re-initialization schemes are unable to re-initialize the function to the SDF. In the proposed formulation, not only is the re-initialization procedure completely eliminated, but the level-set function *ϕ* no longer needs to be initialized as an SDF. The initial level set function *ϕ*_0_ is defined as:

$$\;\phi \left(x,t=0\right)=\left\{\begin{array}{cc} -\rho , & \;x\in \Omega_{0}-\partial \Omega_{0}\\ 0, & \;x\in \partial \Omega_{0}\\ \rho , & \;x\in \Omega -\Omega_{0} \end{array}\right.$$ (43)

where *ρ* is a positive constant, Ω_0_ is the inner region of the initial contour, Ω is the image domain and ∂Ω_0_ refers to the initial contour. The stages of the proposed method can be summarized as:

1. Initialize the level-set function *ϕ* to *ϕ*_0_ using (Eq 43) and the bias field to *b*(*x*) = 0.
2. Compute the edge indicator function *g*(*I*) using (Eq 28).
3. Compute the local intensity means, *c*_1_, *c*_2_, and the global means, *m*_1_, *m*_2_, using Eqs (19) and (6), respectively. Compute the bias field *b*(*x*) from (Eq 18).
4. Calculate *L*_*SPF*_(*I*) and *G*_*SPF*_(*I*) using Eqs (34) and (35), respectively.
5. Solve the partial differential equation (PDE) of *ϕ* using (Eq 36).
6. Regularize the level-set function *ϕ* at time *t* by applying a Gaussian kernel *G*_*χ*_, i.e. *ϕ* = *G*_χ_*ϕ, where *χ* is the standard deviation of the regularizing Gaussian kernel.
7. Check whether the regularized level-set function is stationary. If not, iterate from step (c).

The steps described above correspond to the two-phase segmentation algorithm. However, for the four-phase algorithm, these steps are replaced with the variables corresponding to two level sets using their respective definitions and solutions.

## Results and comparisons

The proposed method was implemented using MATLAB and run on a 3.4 GHz Intel Core-i7 with 16 GB of RAM, testing it on both synthetic images and real brain magnetic resonance (MR) images of 250 × 250 pixels with 256 grey levels (8bpp). The parameters used for all experiments in this section are shown in Table 1.

**Table 1:** Parameters for the experiments in result and comparison section.

	Force term scaling constants		Length term scaling constants	Area term scaling constants	Gaussian kernel tuning constant		Penalizing term scaling constants	Constant for initial level-set	Constant for Heaviside and Dirac Functions	Time step
	*λ*_1_	*λ*_2_	*μ*	*v*	*σ* or *σ*_1_	*χ* or *σ*_2_	*γ*	*ρ*	*ϵ*	Δ*t*
**LBF**	1	1	0.001 × 255^2^	-	4	-	1	1	1.5	0.1
**LIF**	-	-	-	-	3	1	-	2	1.5	1
**LGD**	1	1	0.001 ×255^2^	30	5	-	1/*μ*	2	1.5	0.1
**VLSBCS**	1	1	0.001 ×255^2^	-	3	-	1	1	1	0.1
**LSACM**	1	1	-	20	3	-	0.1	1	1.5	1
**Proposed**	1	5	1	0.25	3	0.5	-	1	1.5	0.1

**Fig 4:**
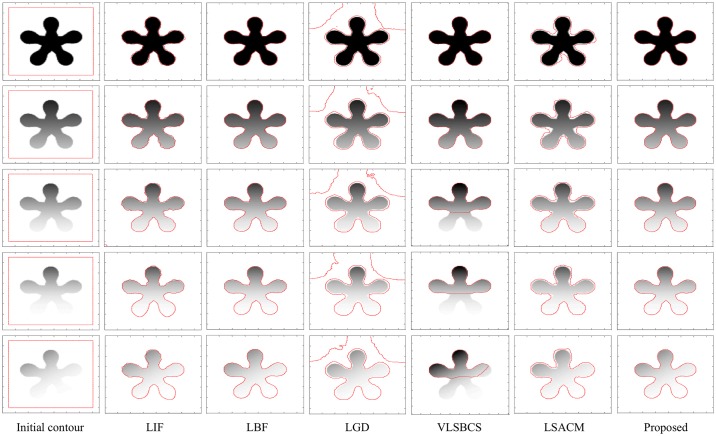
Intensity inhomogeneous image segmentation and comparison with state-of-the-art methods by using images with intensity varying objects.

Fig 4 shows the result of the proposed segmentation method and the comparison with the different state-of-the-art methods that have been tested. In this experiment, we used an image with a single homogeneous object and then progressively changed its intensity distribution to a point at which it is even difficult to manually segment it, thus making the object inhomogeneous.

The first column shows the five input images with the initial contour, whereas the segmentation results are shown using LIF [30] in the second column, LBF [27, 28] in the third column, LGD [29] in the fourth column, VLSBCS [9, 10] in the fifth column, LSACM [11, 12] in the sixth column and the proposed method in the last column, respectively. Visual inspection clearly shows that both the proposed method and LBF provide the best segmentation results. LIF also yields acceptable segmentation results, although the final contour in this method is not quite smooth along the object boundaries. Segmentation results of both LSACM and LGD show that they are not able to strictly find the object boundary. Moreover, the contour in LGD is also stuck in the background, which is undesirable. VLSBCS properly segmented the first two images, but its segmentation results are not acceptable for the last three images. In LGD, *σ* was set to 10 for all experiments in Fig 4, since with a small value of *σ*, this method is unable to segment the objects in all images. For all the examples in Fig 4, the parameters of all methods were kept constant. The only change is the initial position of the level set.

**Fig 5:**
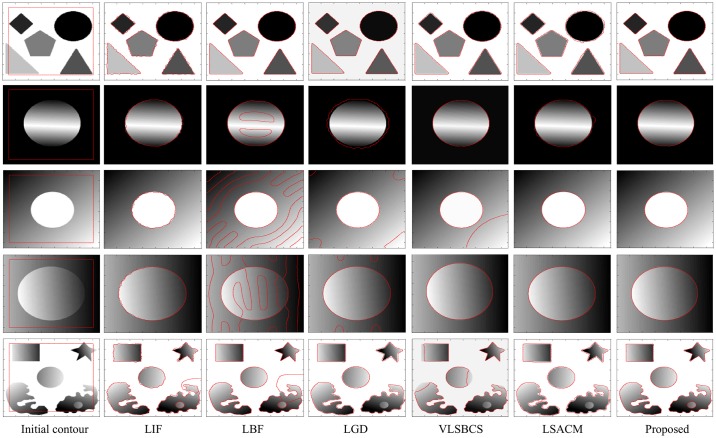
Segmentation results using images with different homogeneity and inhomogeneity possibilities.

Fig 5 shows the experiments conducted with synthetic images with different types of region properties. The first column shows the input images with their respective initial contours. In the first row, both the object and the background are homogeneous. In the second row, the background is homogeneous while the object is inhomogeneous. In the third row, the background is inhomogeneous while the object is homogeneous. In the fourth row, both the background and the object are inhomogeneous. In the last row, the background is homogeneous and there are different inhomogeneous regions with one of the regions having an extra inhomogeneous region within it.

The second column shows the segmentation results with LIF [30]. Notice that it is not able to accurately segment the last image. Although this method is able the segment the objects in the first four images, the results are not acceptable because the contour is not quite smooth along the boundaries of the objects. The segmentation results with LBF [27, 28] are shown in the third column. This method is only able to properly segment the first image, while the segmentation results are not acceptable for the other images. The fourth column shows the segmentation results using LGD [29]. This method is able to properly segment the first image. As for the second image, the contour is not quite smooth along the boundary of the object. Although it is able to segment the objects in the third and fourth images, some undesirable contour is stuck in the background. Furthermore, this method is not able to segment the nested inhomogeneous object in the fifth image.

The fifth column shows the segmentation results using VLSBCS [9, 10]. This method is able to properly segment the first, second and fourth images. It can segment the object in third image. However, some undesirable contour is stuck in the background. Moreover, this method is not able to properly segment the fifth image. The sixth column shows the results with LSACM [11, 12]. This method is able to properly segment the third and fourth images. Although it is able to segment the objects in the first and second images, the final contour is not smooth and does not properly follow the object boundaries. Moreover, this method is unable to segment the nested inhomogeneous object in the fifth image. The last column shows the segmentation results using the proposed method, which is able to properly segment all images.

Table 2 shows a time complexity analysis in terms of CPU time and iterations. The proposed method yields the lowest time complexity for the examples shown in rows 1, 2 and 4, 5. It took 1.58 and 2.19 seconds for the examples shown in the first two rows, respectively. In turn, it took 5.09 and 5.78 seconds for the examples shown in rows 4 and 5, respectively. On the other hand, VLSBCS yields the lowest time complexity for the example shown in row 3. It took 2.27 seconds while the proposed method took 4.17 seconds to obtain the final contour. Although VLSBCS yields the lowest time complexity for this example it is unable to properly segment the object as shown in Fig 4.

**Table 2:** Iterations and CPU time for the examples shown in Fig 4.

Methods		Fig 4				
		Row 1	Row 2	Row 3	Row 4	Row 5
**LIF**	Iterations	400	450	600	2000	2000
	CPU time (s)	5.45	5.97	7.56	57.38	55.91
**LBF**	Iterations	500	1000	1500	2000	2000
	CPU time (s)	7.72	14.38	26.36	32.63	32.44
**LGD**	Iterations	500	500	500	500	500
	CPU time (s)	34.03	32.77	33.69	32.06	34.12
**VLSBCS**	Iterations	20	30	30	100	100
	CPU time (s)	1.71	2.21	**2.27**	5.87	6.31
**LSACM**	Iterations	40	40	50	60	80
	CPU time (s)	24.69	25.55	32.05	38.69	50.72
**Proposed**	Iterations	20	30	70	90	100
	CPU time (s)	**1.58**	**2.19**	4.12	**5.09**	**5.78**

**Fig 6:**
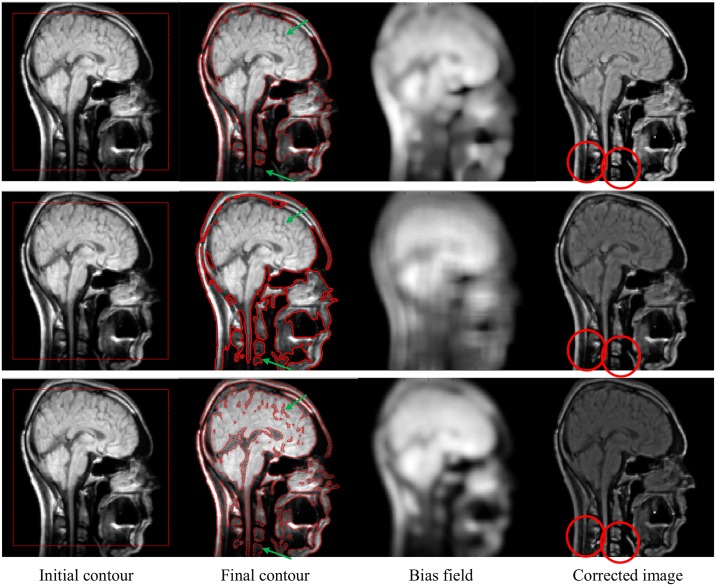
Segmentation and bias correction using Li et al. [9, 10] (first row), Zhang et al. [11, 12] (second row) and the proposed method (third row).

In Fig 6, the segmentation and bias correction results using the proposed method (third row) are compared with the ones computed with VLSBCS [9, 10] (first row) and LSACM [11, 12] (second row) using real brain MR images as a practical application. The first column shows the input image with the initial contour. The second column shows the final contours for each method. The third column shows the estimated bias field. Finally, the last column shows the bias-corrected image. The proposed method can segment more detailed regions than the other methods, as highlighted by the green arrows in the second column. Moreover, the bias-corrected image using the proposed method has more details than the ones computed with the other methods, as highlighted by the red circles in the fourth column. The corrected image obtained using the proposed method also has more details in the nose area.

**Fig 7:**
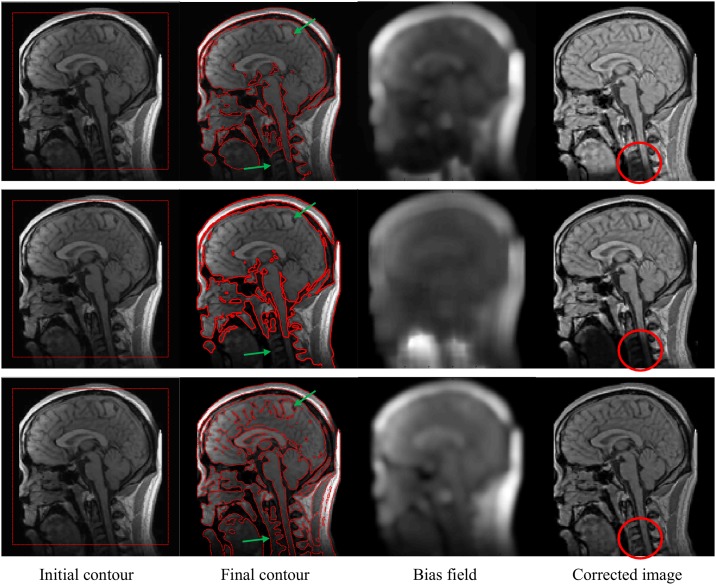
Segmentation and bias correction using Li et al. [9, 10] (first row), Zhang et al. [11, 12] (second row) and the proposed method (third row).

In Fig 7, the segmentation results and bias correction using the proposed method (third row) are compared with the ones computed with VLSBCS [9, 10] (first row) and LSACM [11, 12] (second row) using real brain MR images. The first column shows the input image with the initial contour. The second column shows the final contours. The third column shows the computed bias field. Finally, the last column shows the bias-corrected image. The results show that the proposed method can segment more detailed regions than the other methods, as highlighted by the green arrows in the second column. Moreover, the bias-corrected image using the proposed method has more details than the ones computed with the other methods, as highlighted by the red circles in the fourth column.

**Fig 8:**
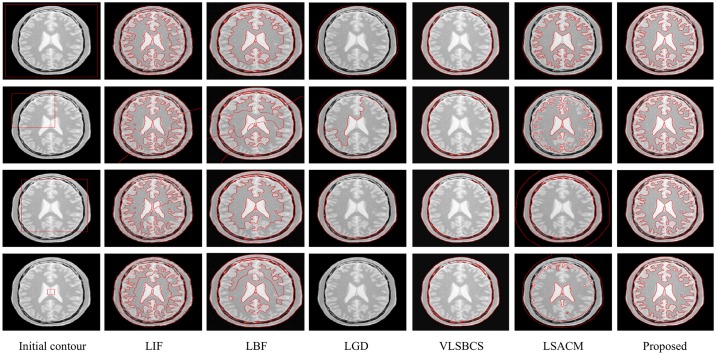
Effect of position of initial contour on the final segmentation results.

Fig 8 shows the effect of the position of the initial level set on the segmentation for all the tested methods. The first column shows the input image with different initial level sets, whereas the segmentation results with LIF [30] are shown in the second column, with LBF [27, 28] in the third column, with LGD [29] in the fourth column, with VLSBCS [9, 10] in the fifth column, with LSACM [11, 12] in the sixth column and with the proposed method in the last column. These results show that the proposed method is not affected by the position of the initial level set and yields the segmentation with the best accuracy. In contrast, the segmentation results of all the tested state-of-the-art methods are affected by the position of the initial level set.

Table 3 shows the number of iterations and CPU time for all the tested methods when applied to the example in Fig 8. The proposed method yields the best performance in the examples shown in rows 2 and 4. It took 2.48 and 1.42 seconds to obtain the final contours for rows 2 and 4, which is significantly lower than the time required by the other methods. In turn, LGD has the best performance for the example in row 1. Thus, LGD took 3.3 seconds while the proposed method took 5.15 seconds to obtain the final contour. In turn, LIF yields the best performance for the example shown in row 3. LIF took 3.86 seconds while the proposed method took 16.06 seconds to obtain the final contour.

**Table 3:** Iterations and CPU time for the examples shown in Fig 8.

Methods		Fig 8			
		Row1	Row2	Row3	Row4
**LIF**	Iterations	250	250	250	200
	CPU time (s)	4.16	4.18	**3.86**	3.29
**LBF**	Iterations	400	400	400	250
	CPU time (s)	8.69	8.18	8.09	5.56
**LGD**	Iterations	200	1500	1000	100
	CPU time (s)	**3.3**	22.91	15.65	2.14
**VLSBCS**	Iterations	100	100	100	100
	CPU time (s)	5.64	7.27	6.47	6.72
**LSACM**	Iterations	250	250	250	600
	CPU time (s)	14.31	14.53	13.98	33.47
**Proposed**	Iterations	80	30	300	10
	CPU time (s)	5.15	**2.48**	16.06	**1.42**

**Fig 9:**
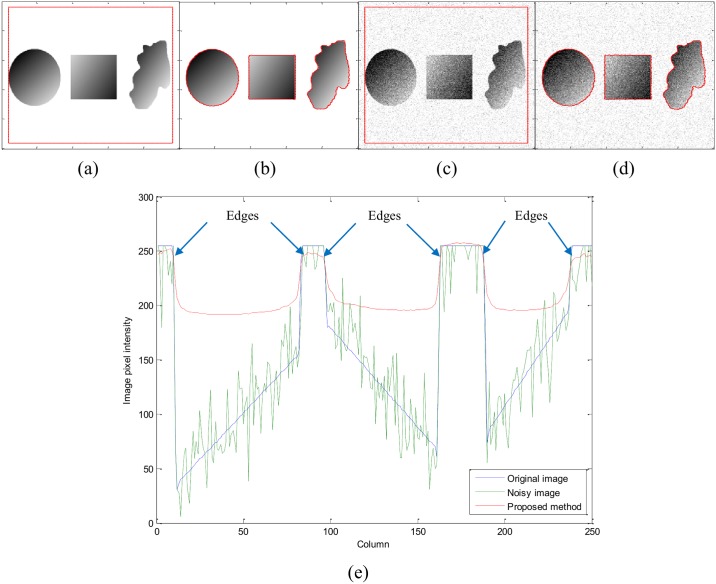
Segmentation results on synthetic images with intensity inhomogeneity with and without noise. (a) Original image with initial contour, (b) Segmentation result using original image without noise, (c) Noisy image with initial contour, (d) Segmentation result using noisy image, (e) Profile selection of the middle rows of the original image (blue line), noisy image (green line) and level set of proposed method (red line).

Fig 9 shows the segmentation result on a synthetic image, which contains three different regions with intensity inhomogeneity, both without noise and after applying additive Gaussian noise. Fig 9(b) and 9(d) show that proposed method is able to properly segment intensity inhomogeneous objects without and with noise, respectively. Fig 9(e) shows the central row intensity profile of the input synthetic image with both clean and noisy data along with the final contour. It shows that the resultant contour followed the object boundaries perfectly.

**Fig 10:**
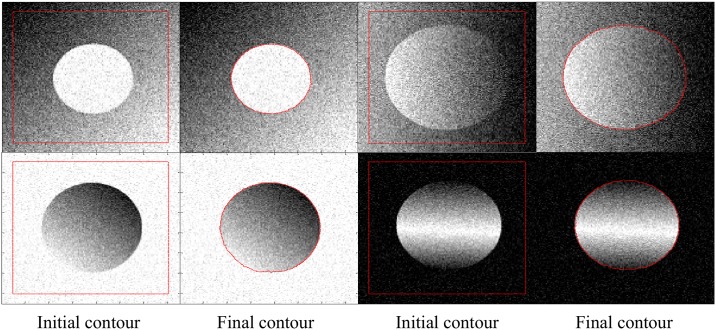
Segmentation results on different intensity inhomogeneous noisy images.

Fig 10 shows some segmentation results by applying the proposed method to different intensity inhomogeneous noisy images. Although noise affected the crispness of edges in the input data, the proposed method is able to yield acceptable segmentation results.

## Quantitative analysis

The segmentation of brain MR images into disjoint regions based on white matter (WM), grey matter (GM) and cerebrospinal fluid (CSF) is a well-known problem in brain image analysis. Due to the geometric complexity of the human brain cortex, manual slice-by-slice segmentation is cumbersome and time consuming [1]. Thus, numerous methods have been devised to solve such problems. Active contours are quite popular in this context. Because of the complex intensity inhomogeneous regions, brain MR images are hard to be successfully segmented with high accuracy [39]. Therefore, this is a good test bench to test the proposed method in a practical application and compare it with other state-of-the-art methods.

### Two-phase active contours

This section shows segmentation results for all tested two-phase active contour methods using 2D brain MR images from a public database of 20 brain anatomical models [37]. All images have 250 × 250 pixels and 8 bits per pixel.

Active contour methods behave differently for different types of images. Because the images used in this section have different characteristics compared to the ones used in results and comparison section, the parameters had to be tuned in order to obtain the best possible segmentation results. The parameters used for all experiments in this section are shown in Table 4.

**Table 4:** Parameters for the experiments in quantitative analysis section.

	Force term scaling constants		Length term scaling constant	Area term scaling constant	Gaussian kernel tuning constant		Penalizing term scaling constant	Constant for initial level-set	Constant for Heaviside and Dirac Functions	Time step
	*λ*_1_	*λ*_2_	*μ*	*v*	*σ* or *σ*_1_	*χ* or *σ*_2_	*γ*	*ρ*	*ϵ*	Δ*t*
**LBF**	1	1	0.001 × 255^2^	-	3	-	1	1	1.5	0.1
**LIF**	-	-	-	-	2	0.5	-	2	1.5	1
**LGD**	1	1	0.001 × 255^2^	30	3	-	1/*μ*	2	1.5	0.1
**VLSBCS**	1	1	0.001 × 255^2^	-	3	-	1	1	1	0.1
**LSACM**	1	1	-	20	3	-	0.1	1	1.5	1
**Proposed**	5	1	1	0.25	3	0.5	-	1	1.5	1

**Fig 11:**
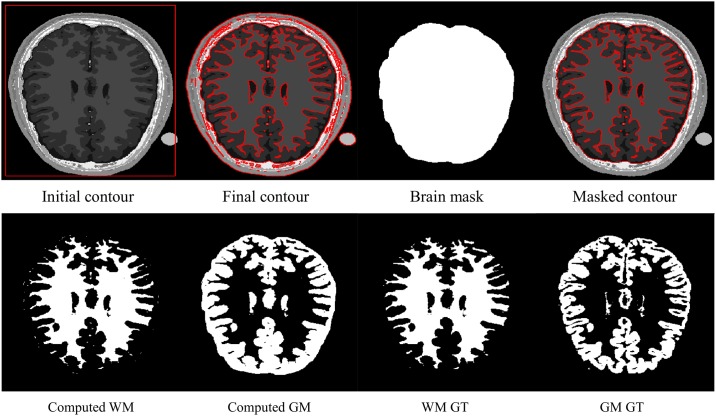
WM and GM regions computed with the proposed method and their respective ground truths.

In order to partition a brain MR image into WM and GM regions, the segmentation result is split into two regions based on two phases: *ϕ*> 0 and *ϕ*< 0. The WM and GM regions represent the brain region, which is the region of interest, while the regions outside the brain (e.g., skull, fat and vacuum) can be taken as irrelevant regions. Therefore, we manually extracted the brain area to segment the WM and GM regions, removing the other irrelevant regions out of the brain. Fig 11 shows the WM and GM regions computed from the two phases of the proposed method and the comparison with the ground truth (GT). In the first row of Fig 11, the first image shows the input image with the initial contour. The second image shows the final contour using the proposed method. The third image shows a manually defined brain mask. The main purpose of the brain mask is to extract the brain region in order to carefully analyse and compare the segmented WM and GM regions with their respective ground truths. The last image in the first row shows the final contour after scaling with the brain mask. Let *ϕ*(*x*, *y*) be the final computed contour shown in the second column of the first row, and *m*(*x*, *y*) the manually defined brain mask shown in the third column of the first row. The scaled final contour *ξ*(*x*, *y*), which is shown in the last column of the first row, is computed as *ξ*(*x*, *y*) = *ϕ*(*x*, *y*)*m*(*x*, *y*). In the second row of Fig 11, the first image shows the WM region computed from the positive phase (*ξ*> 0) of the final contour scaled with the brain mask. The second image shows the GM region computed using the negative phase (*ξ*< 0) of the scaled final contour. In the second row, the third and fourth images show the ground truths of the WM and GM regions, respectively.

**Fig 12:**
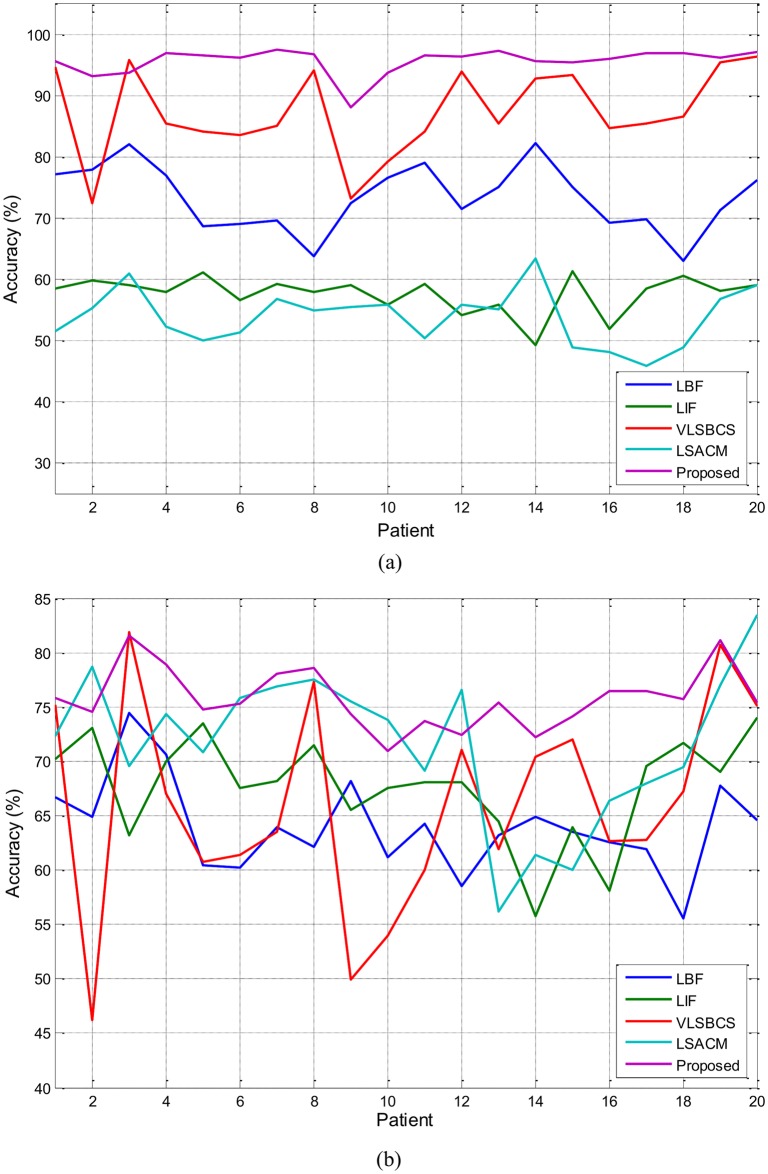
Segmentation accuracy analysis of (a) WM and (b) GM regions using two-phase active contours.

Fig 12 shows the accuracy analysis of the region of interest in the brain MR images. A total of 100 2D slices from 20 brain anatomical models [37] were used. Five 2D slices from every patient were considered. The WM and GM regions for all methods were computed as depicted in Fig 11. The segmentation accuracy corresponding to the WM and GM regions presented in Fig 12 was obtained as:

$$\;\text{Accuracy}=\frac{\left|A\cap B\right|}{\left|A\cup B\right|}\times 100,$$ (44)

Where *A* is the computed WM or GM region after brain scaling and *B* is the ground truth for that region. Different methods can behave differently based on the type of images. Therefore, five slices from each patient were used and average accuracies for those slices were computed to obtain a representative value for each case. Fig 12 and Table 5 show that the proposed method yields the best segmentation accuracy in most cases for both the WM and GM regions.

**Table 5:** Segmentation accuracy of WM and GM regions using the two-phase active contour methods.

Regions	LBF	LIF	VLSBCS	LSACM	Proposed
**WM**	73.34	57.65	87.32	53.82	**95.69**
**GM**	63.97	67.64	66.04	71.63	**75.78**

### Four-phase active contours

A two-phase method is limited to segmenting images into two regions, which is its big weakness in its application to brain MR image segmentation. In the last subsection, all of the two-phase tested methods are evaluated for WM and GM region segmentation. However, the segmented GM region was a combination of GM and CSF regions, resulting into a high amount of false positives. In this subsection, the four-phase model of the proposed active contour method is evaluated along with the state-of-the-art four-phase active contour methods with respect to the segmentation accuracy of WM, GM and CSF regions. All methods are tested using the same brain MR image database mentioned in the previous subsection with the same image size and intensity. The parameters used for the proposed method are: *λ*_1_ = 2, *λ*_2_ = 2, *μ* = 5, *σ* = 3, *χ* = 0.45, *ρ* = 1 *ϵ* = 1.5 and Δ*t* = 1.

In brain MR images, WM, GM and CSF regions represent the brain, which is the region of interest. The regions outside the brain (e.g., skull, fat and vacuum) can be assumed to be irrelevant regions. In order to remove those unwanted regions, it is necessary to strip the skull from the brain MR image using a manually drawn brain mask. A brain mask is used as shown in Fig 11, although it is used for the four-phase method. Let *ϕ*_1_(*x*, *y*) and *ϕ*_2_(*x*, *y*) be the final computed contours for two level sets, *m*(*x*, *y*) be the manually defined brain mask and *ξ*_1_(*x*, *y*) and *ξ*_2_(*x*, *y*) scaled final contours with the brain mask which are computed as: *ξ*_1_(*x*, *y*) = *ϕ*_1_(*x*, *y*)*m*(*x*, *y*) and *ξ*_2_(*x*, *y*) = *ϕ*_2_(*x*, *y*)*m*(*x*, *y*). By using the proposed method, the four regions are computed as follows: *R*_1_ = (*ξ*_1_> 0) ∩ (*ξ*_2_> 0), *R*_2_ = (*ξ*_1_> 0) ∩ (*ξ*_2_< 0), *R*_3_ = (*ξ*_1_< 0) ∩ (*ξ*_2_> 0) and *R*_4_ = (*ξ*_1_< 0) ∩ (*ξ*_2_< 0). In brain MR images, *R*_1_, *R*_2_ and *R*_3_regions usually refer to WM, GM and CSF regions, respectively, and *R*_4_ is an empty region, which is discarded.

**Fig 13:**
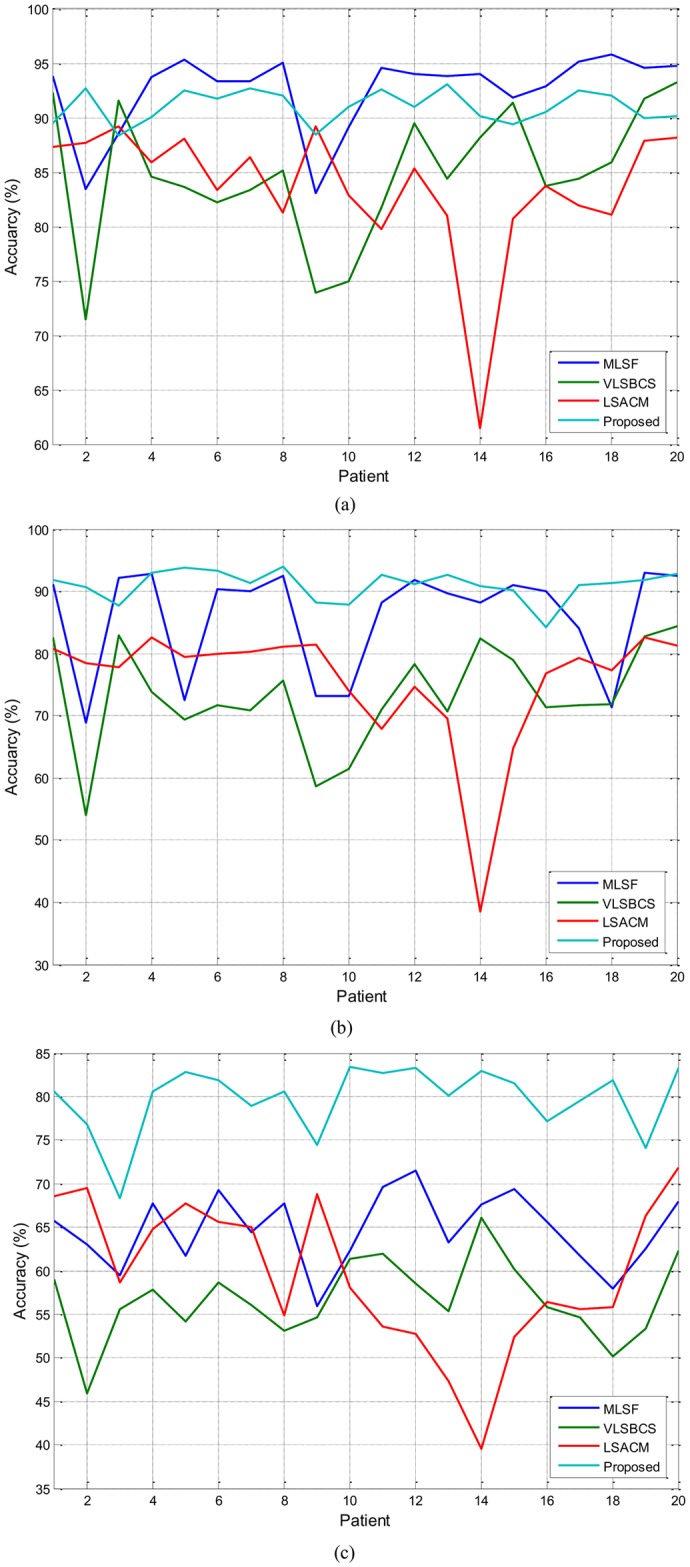
Segmentation accuracy analysis of (a) WM (b) GM and (c) CSF regions using four-phase active contours.

Fig 13 shows the accuracy analysis of the regions of interest (i.e., WM, GM and CSF regions) in the brain MR images. A total of 100 2D slices from 20 brain anatomical models [37] were used. Five 2D slices (namely 150, 175, 200, 225 and 250) from every patient were considered. The WM, GM and CSF regions are segmented using MLSF, VLSBCS, LSACM and the proposed methods. The accuracy of WM, GM and CSF regions for the mentioned four-phase active contour methods are computed using (44). In (44), *A* is the computed WM, GM or CSF region and *B* is the respective ground truth.

Fig 13 and Table 6 show that the proposed method yields the best segmentation results for GM and CSF regions. In turn, MLSF method yields the best segmentation results for the WM region with an accuracy of 92.52%. The proposed method yields an accuracy of 91.02% for the WM region, which is 1.5% less than the one with the MLSF method. LSACM and VLSBCS methods yield unsatisfactory segmentation results compared to both the proposed and the MLSF method. Table 6 also shows the CPU time comparison between the evaluated methods. It shows that MLSF is the quickest among the compared four-phase active contour methods. It took an average of 15.12 seconds to get the final segmentation result. In turn, the proposed method took an average of 19.01 seconds to fully converge, which is approximately 4 seconds more than the MLSF method.

**Table 6:** Segmentation accuracy of WM, GM and CSF regions using the four-phase active contour methods.

Methods	WM	GM	CSF	CPU time (s)
**MLSF**	**92.52**	85.84	64.71	**15.12**
**VLSBCS**	84.88	73.22	56.73	18.33
**LSACM**	83.62	75.43	59.66	91.30
**Proposed**	91.02	**91.00**	**79.73**	19.01

### Comparison with brain MR image segmentation softwares

**Fig 14:**
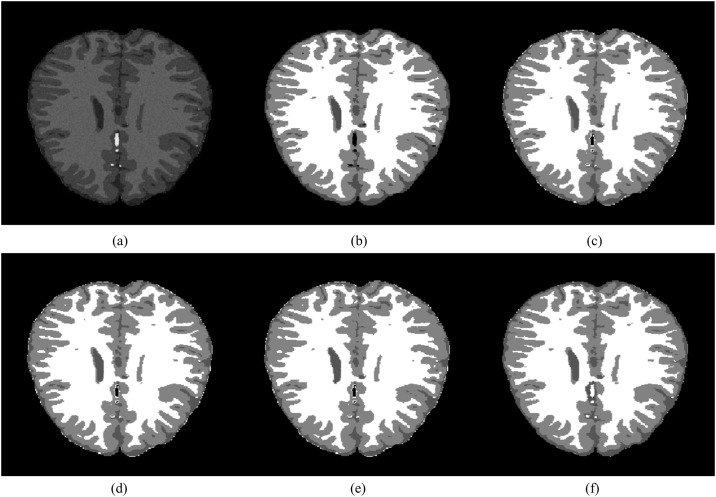
Visual comparison of the segmentation results of the proposed method with alternative brain MR image segmentation softwares. (a) Skull stripped brain image, (b) Ground truth (c) SPM, (d) FSL, (e) BrainSuite and (f) Proposed method.

In this section, the segmentation results of the proposed method are compared with three alternative open source brain MR image segmentation softwares: SPM [40, 41], LSF [42, 43] and BrainSuite [44, 45] using the Brain Web database. In order to compare the segmentation results, the brain region was extracted by stripping the skull using a brain mask. Fig 14 shows a skull stripped brain region and the visual comparison of the segmented regions with their respective ground truth. It shows that all the compared methods yield similar segmentation results from a qualitative point of view.

Table 7 shows the segmentation accuracy of the evaluated methods using two similarity metrics. Both the mean and standard deviation of the evaluated metrics are considered for all three brain regions, i.e., WM, GM and CSF. These similarity metrics are the Jaccard index [46] and the Dice coefficient [47], which are frequently used when the ground truth of the regions of interest is available. These similarity metrics are defined as:

$$\begin{array}{ll} J\left(S,G\right) & =\frac{\left|S\cap G\right|}{\left|S\cup G\right|},\\ D\left(S,G\right) & =\frac{2\left|S\cap G\right|}{\left|S\right|+\left|G\right|} \end{array}$$ (45)

where *S* is the segmented region and *G* its respective ground truth.

**Table 7:** Segmentation accuracy of WM, GM and CSF regions for the proposed method and the evaluated brain segmentation softwares.

Regions	Brain segmentation softwares	Dice coefficient	Jaccard index
**WM**	SPM	0.95±0.02	0.90±0.03
	FSL	0.93±0.02	0.86±0.03
	BrainSuite	0.91±0.06	0.84±0.09
	Proposed	**0.96±0.01**	**0.92±0.02**
**GM**	SPM	0.93±0.02	0.87±0.04
	FSL	0.92±0.02	0.85±0.04
	BrainSuite	0.80±0.16	0.68±0.20
	Proposed	**0.94±0.01**	**0.88±0.02**
**CSF**	SPM	0.76±0.02	0.61±0.02
	FSL	0.74±0.06	0.60±0.02
	BrainSuite	0.46±0.16	0.31±0.08
	Proposed	**0.88±0.01**	**0.80±0.02**

Table 7 shows that the proposed method yields the best segmentation accuracy using both similarity measures for all three brain regions, i.e., WM, GM and CSF. For the GM region, if we consider the mean error (standard deviation) then the Dice coefficient computed using SPM is 0.95, which is the same as the proposed method. However, SPM yields a Jaccard index of 0.91, which is 0.01 more than the Jaccard index computed by the proposed method.

## Conclusion

This paper proposes a new region-based active contour method for image segmentation and bias correction by using an energy functional based on both local and global fitted images. In order to minimize that energy functional, the square image fitted difference is formulated by using The energy functionalboth local and global fitted differences. In the gradient descent solution, both the local and global image differences are replaced by local and global signed pressure force (SPF) functions. Finally, a Gaussian kernel is applied to regularize the curve at each step and avoid the computationally expensive re-initialization.

The main contribution of this paper is the formulation of a new energy functional from the LIF method [30] and the modification of the attained gradient descent solution with new SPF functions based on local and global fitted images to make the solution more stable. Qualitative and quantitative analysis show that the proposed method yields significantly better segmentation results and correction of homogeneous regions than alternative state-of-the-art methods. MR images have been used as a practical test bench.

One of the drawbacks of a local fitted region-based active contour method is its high time complexity. In future work, we aim to formulate a new region-based active contour method by modifying the proposed energy functional in a way to reduce time complexity. In order to do that, we plan to integrate a phase-shift approach similar to the one proposed in [48].

## Appendix

**A. Derivation of the gradient descent flow of the two-phase model** In (Eq 25), the variation *η* is added to the level-set function *ϕ* such that $\phi =\tilde{\phi }+\epsilon \eta$. Keeping *c*_1_, *c*_2_, *m*_1_, *m*_2_ and *b*(*x*) fixed, differentiating with respect to *ϕ* and letting *ϵ*→ 0, we have:

$$\;\begin{array}{ll} \frac{\partial E_{LGFI}}{\partial \varphi } & =\underset{\epsilon \to 0}{\text{lim}}\frac{d}{d\epsilon }(\frac{1}{2}\int_{\Omega }(I-b(c_{1}H_{\epsilon }(\tilde{\phi })+c_{2}(1-H_{\epsilon }(\tilde{\phi }))))\\ & \left(I-(m_{1}H_{\epsilon }(\tilde{\phi })+m_{2}(1-H_{\epsilon }(\tilde{\phi }))))dx\right)\\ & =\frac{1}{2}\underset{\epsilon \to 0}{\text{lim}}(-\int_{\Omega }[(I-b(c_{1}H_{\epsilon }(\tilde{\phi })+c_{2}(1-H_{\epsilon }(\tilde{\phi }))))(m_{1}-m_{2})\\ & -b(I-(m_{1}H_{\epsilon }(\tilde{\phi })+m_{2}(1-H_{\epsilon }(\tilde{\phi }))))(c_{1}-c_{2})]\delta_{\epsilon }(\tilde{\phi })\eta dx)\\ & =\frac{1}{2}\underset{\epsilon \to 0}{\text{lim}}(-\int_{\Omega }[(I-b(c_{1}H_{\epsilon }(\phi )+c_{2}(1-H_{\epsilon }(\phi ))))(m_{1}-m_{2})\\ & -b(I-(m_{1}H_{\epsilon }(\phi )+m_{2}(1-H_{\epsilon }(\phi ))))(c_{1}-c_{2})]\delta_{\epsilon }(\phi )\eta dx)\; \end{array}\;$$

The following Euler Lagrange equation is obtained:

$$\begin{array}{l} -[\left(I-b\left(c_{1}H_{\epsilon }\left(\phi \right)+c_{2}\left(1-H_{\epsilon }\left(\phi \right)\right)\right)\right)\left(m_{1}-m_{2}\right)\\ +b\left(I-\left(m_{1}H_{\epsilon }\left(\phi \right)+m_{2}\left(1-H_{\epsilon }\left(\phi \right)\right)\right)\right)\left(c_{1}-c_{2}\right)]\delta_{\epsilon }\left(\phi \right)=0 \end{array}$$

By applying steepest gradient descent [38], the final gradient descent flow is obtained:

$$\begin{array}{ll} \frac{\partial \phi }{\partial t} & =[(I-b(c_{1}H_{\epsilon }(\phi )+c_{2}(1-H_{\epsilon }(\phi ))))(m_{1}-m_{2})\\ & +b(I-(m_{1}H_{\epsilon }(\phi )+m_{2}(1-H_{\epsilon }(\phi ))))(c_{1}-c_{2})]\delta_{\epsilon }(\phi )\\ & =((I-I_{{\text{​}}_{bLFI}})(m_{1}-m_{2})+b(I-I_{{\text{​}}_{GFI}})(c_{1}-c_{2}))\delta_{\epsilon }(\phi ) \end{array}$$

**B. Derivation of the gradient descent flow of the four-phase model** By using the definitions of Eqs (33) and (34) in (Eq 25), and adding the variations *η*_1_ and *η*_2_ to the level set functions *ϕ*_1_ and *ϕ*_2_, respectively, such that $\tilde{\phi_{1}}=\phi_{1}+\epsilon \eta_{1}$ and $\tilde{\phi_{2}}=\phi_{2}+\epsilon \eta_{2}$. Keeping *c*_1_, *c*_2_, *c*_3_, *c*_4_, *m*_1_, *m*_2_, *m*_3_, *m*_4_ and *ϕ*_2_ fixed, differentiating with respect to *ϕ*_1_and letting *ϵ*→ 0, we have:

$$\begin{array}{ll} \frac{\partial E_{LGFI}}{\partial \phi } & =\underset{\epsilon \to 0}{\text{lim}}\frac{d}{d\epsilon }(\frac{1}{2}\int_{\Omega }(I-b(c_{1}H_{\epsilon }(\tilde{\phi_{1}})H_{\epsilon }(\tilde{\phi_{2}})+c_{2}H_{\epsilon }(\tilde{\phi_{1}})(1-H_{\epsilon }(\tilde{\phi_{2}}))\\ & +c_{3}(1-H_{\epsilon }(\tilde{\phi_{1}}))H_{\epsilon }(\tilde{\phi_{2}})+c_{4}(1-H_{\epsilon }(\tilde{\phi_{1}}))(1-H_{\epsilon }(\tilde{\phi_{2}}))))\\ & \left(I-(m_{1}H_{\epsilon }(\tilde{\phi_{1}})H_{\epsilon }(\tilde{\phi_{2}})+m_{2}H_{\epsilon }(\tilde{\phi_{1}})(1-H_{\epsilon }(\tilde{\phi_{2}})\right)\\ & +m_{3}(1-H_{\epsilon }(\tilde{\phi_{1}}))H_{\epsilon }(\tilde{\phi_{2}})+m_{4}(1-H_{\epsilon }(\tilde{\phi_{1}}))(1-H_{\epsilon }(\tilde{\phi_{2}}))))dx)\\ & =\frac{1}{2}\underset{\epsilon \to 0}{\text{lim}}(\int_{\Omega }-b(I-(m_{1}H_{\epsilon }(\tilde{\phi_{1}})H_{\epsilon }(\tilde{\phi_{2}})+m_{2}H_{\epsilon }(\tilde{\phi_{1}})(1-H_{\epsilon }(\tilde{\phi_{2}}))\\ & +m_{3}(1-H_{\epsilon }(\tilde{\phi_{1}}))H_{\epsilon }(\tilde{\phi_{2}})+m_{4}(1-H_{\epsilon }(\tilde{\phi_{1}}))(1-H_{\epsilon }(\tilde{\phi_{2}}))))\\ & \left((c_{1}-c_{3})H_{\epsilon }(\tilde{\phi_{2}})+(c_{2}-c_{4})(1-H_{\epsilon }(\tilde{\phi_{2}}))\right)\\ & -(I-b(c_{1}H_{\epsilon }(\tilde{\phi_{1}})H_{\epsilon }(\tilde{\phi_{2}})+c_{2}H_{\epsilon }(\tilde{\phi_{1}})(1-H_{\epsilon }(\tilde{\phi_{2}}))\\ & +c_{3}(1-H_{\epsilon }(\tilde{\phi_{1}}))H_{\epsilon }(\tilde{\phi_{2}})+c_{4}(1-H_{\epsilon }(\tilde{\phi_{1}}))(1-H_{\epsilon }(\tilde{\phi_{2}}))))\\ & \left((m_{1}-m_{3})H_{\epsilon }(\tilde{\phi_{2}})+(m_{2}-m_{4})(1-H_{\epsilon }(\tilde{\phi_{2}})))\delta_{\epsilon }(\tilde{\phi_{1}})\eta_{1}dx\right)\\ & =\frac{1}{2}\underset{\epsilon \to 0}{\text{lim}}(\int_{\Omega }-b(I-(m_{1}H_{\epsilon }(\phi_{1})H_{\epsilon }(\phi_{2})+m_{2}H_{\epsilon }(\phi_{1})(1-H_{\epsilon }(\phi_{2}))\\ & +m_{3}(1-H_{\epsilon }(\phi_{1}))H_{\epsilon }(\phi_{2})+m_{4}(1-H_{\epsilon }(\phi_{1}))(1-H_{\epsilon }(\phi_{2}))))\\ & \left((c_{1}-c_{3})H_{\epsilon }(\phi_{2})+(c_{2}-c_{4})(1-H_{\epsilon }(\phi_{2}))\right)\\ & -(I-b(c_{1}H_{\epsilon }(\phi_{1})H_{\epsilon }(\phi_{2})+c_{2}H_{\epsilon }(\phi_{1})(1-H_{\epsilon }(\phi_{2}))\\ & +c_{3}(1-H_{\epsilon }(\phi_{1}))H_{\epsilon }(\phi_{2})+c_{4}(1-H_{\epsilon }(\phi_{1}))(1-H_{\epsilon }(\phi_{2}))))\\ & \left((m_{1}-m_{3})H_{\epsilon }(\phi_{2})+(m_{2}-m_{4})(1-H_{\epsilon }(\phi_{2})))\delta_{\epsilon }(\phi_{1})\eta_{1}dx\right) \end{array}$$

The following Euler Lagrange equation is obtained:

$$\begin{array}{l} -[b(I-(m_{1}H_{\epsilon }(\phi_{1})H_{\epsilon }(\phi_{2})+m_{2}H_{\epsilon }(\phi_{1})(1-H_{\epsilon }(\phi_{2}))+m_{3}(1-H_{\epsilon }(\phi_{1}))H_{\epsilon }(\phi_{2})\\ +m_{4}(1-H_{\epsilon }(\phi_{1}))(1-H_{\epsilon }(\phi_{2}))))((c_{1}-c_{3})H_{\epsilon }(\phi_{2})+(c_{2}-c_{4})(1-H_{\epsilon }(\phi_{2})))\\ +(I-b(c_{1}H_{\epsilon }(\phi_{1})H_{\epsilon }(\phi_{2})+c_{2}H_{\epsilon }(\phi_{1})(1-H_{\epsilon }(\phi_{2}))+c_{3}(1-H_{\epsilon }(\phi_{1}))H_{\epsilon }(\phi_{2})\\ +c_{4}(1-H_{\epsilon }(\phi_{1}))(1-H_{\epsilon }(\phi_{2}))))((m_{1}-m_{3})H_{\epsilon }(\phi_{2})\\ +(m_{2}-m_{4})(1-H_{\epsilon }(\phi_{2}))]\delta_{\epsilon }(\phi_{1})=0 \end{array}$$

By using the steepest gradient descent method [38], the final gradient descent flow obtained for *ϕ*_1_ is:

$$\begin{array}{ll} \frac{\partial \phi_{1}}{\partial t} & =[b(I-(m_{1}H_{\epsilon }(\phi_{1})H_{\epsilon }(\phi_{2})+m_{2}H_{\epsilon }(\phi_{1})(1-H_{\epsilon }(\phi_{2}))+m_{3}(1-H_{\epsilon }(\phi_{1}))H_{\epsilon }(\phi_{2})\\ & +m_{4}(1-H_{\epsilon }(\phi_{1}))(1-H_{\epsilon }(\phi_{2}))))((c_{1}-c_{3})H_{\epsilon }(\phi_{2})+(c_{2}-c_{4})(1-H_{\epsilon }(\phi_{2})))\\ & +(I-b(c_{1}H_{\epsilon }(\phi_{1})H_{\epsilon }(\phi_{2})+c_{2}H_{\epsilon }(\phi_{1})(1-H_{\epsilon }(\phi_{2}))+c_{3}(1-H_{\epsilon }(\phi_{1}))H_{\epsilon }(\phi_{2})\\ & +c_{4}(1-H_{\epsilon }(\phi_{1}))(1-H_{\epsilon }(\phi_{2}))))((m_{1}-m_{3})H_{\epsilon }(\phi_{2})\\ & +(m_{2}-m_{4})(1-H_{\epsilon }(\phi_{2}))]\delta_{\epsilon }(\phi_{1})\\ & =[b(I-I_{GFI})((c_{1}-c_{3})H_{\epsilon }(\phi_{2})+(c_{2}-c_{4})(1-H_{\epsilon }(\phi_{2})))\\ & +(I-I_{bLFI})((m_{1}-m_{3})H_{\epsilon }(\phi_{2})+(m_{2}-m_{4})(1-H_{\epsilon }(\phi_{2}))]\delta_{\epsilon }(\phi_{1}) \end{array}$$

Similarly, keeping *c*_1_, *c*_2_, *c*_3_, *c*_4_, *m*_1_, *m*_2_, *m*_3_, *m*_4_and *ϕ*_1_fixed, differentiating with respect to *ϕ*_2_and by the steepest gradient descent method [38], the following gradient descent flow for *ϕ*_2_ is obtained:

$$\begin{array}{ll} \frac{\partial \phi_{2}}{\partial t} & =[b\left(I-I_{GFI}\right)\left(\left(c_{1}-c_{2}\right)H_{\epsilon }\left(\phi_{1}\right)+\left(c_{3}-c_{4}\right)\left(1-H_{\epsilon }\left(\phi_{1}\right)\right)\right)\\ & +\left(I-I_{bLFI}\right)\left(\left(m_{1}-m_{2}\right)H_{\epsilon }\left(\phi_{1}\right)+\left(m_{3}-m_{4}\right)\left(1-H_{\epsilon }\left(\phi_{1}\right)\right)\right]\delta_{\epsilon }\left(\phi_{2}\right) \end{array}$$

## Supporting information

S1 Dataset Contains synthetic and real brain MR images used in Figs 1, 4, 5, 6, 7, 8 and 9. (RAR)

S2 Dataset Contains .mat files of brain masks used for image segmentation. (RAR)

S1 Supporting Information Contains MATLAB code of the two-phase active contours formulation. (RAR)

S2 Supporting Information Contains MATLAB code of the four-phase active contours formulation. (RAR)
